# Emplacement of trauma in migrant spaces: non-places and unhomeliness in *Living Undocumented* and *Stateless*

**DOI:** 10.3389/fsoc.2025.1619372

**Published:** 2025-07-24

**Authors:** Angel Treesa Joseph, Rashmi Rekha Borah

**Affiliations:** Social Sciences and Languages, Vellore Institute of Technology, Chennai, India

**Keywords:** non-place, unhomeliness, trauma, migration, dislocation

## Abstract

Migration often places individuals into spaces that lack stability, familiarity, and personal connection. This paper investigates the emplacement of trauma in non-places, focusing on the experiences of immigrants as depicted in the Netflix series *Living Undocumented* (2019) and *Stateless* (2020). Utilizing the theoretical frameworks of Marc Augé’s non-places and Homi Bhabha’s concept of unhomeliness, the study analyzes how these liminal spaces strip migrants of their identity, destabilize their sense of belonging, and intensify their emotional and psychological distress. The analysis examines how non-places contribute to feelings of dislocation, alienation, and cultural trauma, intensifying the struggles associated with identity and belonging in unfamiliar territories. By analyzing the interaction between individuals and these non-spaces, the paper highlights a complex layer of emotional and cultural estrangement often overlooked in discussions of migration. The article argues for a re-evaluation of immigration policies, advocating for approaches that emphasize empathy, inclusion, and respect for human dignity. This research contributes to the evolving discourse on migration, identity, and space, calling for a paradigmatic shift in perspective on how non-places impact the lives of migrants.

## 1 Introduction

I want you to imagine waking up one morning and your father is just gone. I want you to imagine going home and trying to tell everybody that everything will be okay when you yourself aren’t even sure of that. And I want you to imagine trying to sleep every night, only to find yourself lying awake for hours because you cannot sleep. That worry will end you. And it will try to break you. (Saidman and Chai, 2019, A Prayer in the Night 0:10).

The opening scene of the Netflix documentary series *Living Undocumented* presents a world where traditional notions of place are upended, leading to a destabilizing sense of placelessness. According to The World Migration Report (2024), the global landscape of migration is becoming increasingly complex, with approximately 281 million international migrants comprising about 3.6% of the world’s population. Increasing numbers of individuals are compelled to relocate within and beyond their home countries due to factors such as conflict, economic or political instability, and environmental disasters (Hammar et al., 2021; Migration Data Portal, 2024; Mitchell and Pizzi, 2020). The gruesome realities of migration, displacement, trauma, and the search for identity are powerfully portrayed in the Netflix documentaries *Living Undocumented* (2019) and *Stateless* (2020). Both series share the stories of migrants as they face the overwhelming challenges of living in countries where they are treated as outsiders and forced to maneuver through unwelcoming systems that threaten their sense of belonging.

*Living Undocumented*, a 2019 Netflix documentary series co-directed by Aaron Saidman and Anna Chai, presents the lives of eight undocumented immigrant families in the United States (Horton, 2019). The series highlights the emotional and psychological impact of the U. S. immigration system on these families, revealing the fear, uncertainty, and hope that define their everyday existence (Klein, 2019). Luis, an asylum seeker from Honduras, alongside his partner Kenia, faces constant deportation fears, mirroring the instability many undocumented immigrants endure. Ron and his wife have spent 17 years in the U. S., sustaining a business through a tax ID, embodying the dedicated immigrants contributing to the economy, yet plagued by the fear of enforcement actions. Alejandra, having resettled after multiple perilous journeys from Mexico, grapples with the irony of her military spouse supporting the political forces threatening her presence in the U. S. The Dunoyer family from Colombia exemplifies the harsh realities leading families to seek asylum amidst disbelief from the systems meant to protect them. Eddie, arriving as a child, now faces significant legal hurdles as an adult due to his undocumented status, and Awa diligently supports her father, a Mauritanian asylum seeker, in his fight against deportation.

*Stateless*, launched on ABC TV and later released on Netflix in 2020, is an Australian drama partially inspired by the real-life story of Cornelia Rau. Created by Cate Blanchett, Tony Ayres, and Elise McCredie, this six-episode series unfolds in an Australian detention center, interweaving the lives of four strangers: a troubled airline hostess, an Afghan refugee, a young father escaping a dead-end job, and a bureaucrat embroiled in a national scandal. Elise McCredie, writer of *Stateless,* notes that the four principal characters including a guard, a bureaucrat from the Department of Immigration, an Afghan refugee, and a mentally ill woman of German descent who is mistakenly held as an undocumented immigrant, were all based on real individuals and actual events (Cooney, 2020).

While a wide range of global media engages with migration and displacement, *Living Undocumented* and *Stateless* were selected for their high visibility, critical reception, and contrasting narrative forms, with one presented as a documentary and the other as dramatized fiction. Both series are products of Anglophone, Western media industries, specifically the United States and Australia, and they demonstrate how mainstream platforms such as Netflix and ABC shape dominant imaginaries of migration, statelessness, and bureaucratic exclusion. However, although the production contexts are Western, the narratives themselves portray migrants from a diverse range of countries, including Mexico, Honduras, Colombia, Laos, Israel, and Jamaica in *Living Undocumented*, and Afghanistan, Iran, Sri Lanka, and Iraq in *Stateless*. These portrayals reflect the structural and emotional realities of global displacement, encompassing a variety of sociopolitical and cultural backgrounds. Furthermore, the article focuses on affective geographies, particularly non-place and unhomeliness, which enables an interpretive framework that is not limited to national policy contexts. Finally, analyzing high-production, widely distributed media allows the study to critically assess how global audiences engage with and understand migrant subjectivities, and how such narratives may both challenge and reproduce dominant systems of exclusion.

In addition to their narrative structures, both series are grounded in firsthand accounts and testimonies of displacement. *Living Undocumented* includes personal stories of migrants facing deportation and legal precarity, which reviewers have described as giving “a human face to immigrant stories” (ReFrame, 2019). Media reports and interviews with executive producer Selena Gomez highlight real-life encounters with individuals such as bar, a teenager from Israel living in fear after experiencing robbery and insecurity (Bruney, 2019; TIME, 2019). Similarly, *Stateless* draws upon documented experiences from Cornelia Rau’s wrongful detention, as well as testimonies from former Afghan detainees, which have been referenced in interviews with the creators and actors (Diandra, 2020; Variety, 2020). These accounts offer an additional layer of empirical grounding that enhances the interpretive framework of this study by connecting the aesthetic and theoretical with the lived and testimonial.

This article examines how *Living Undocumented* and *Stateless* employs the concepts of non-place and unhomeliness to portray the trauma and uncertainty that shape the migrant experience. The theoretical overview section explores current research on the concepts of non-places and unhomeliness, particularly drawing on the work of Marc Augé and Homi Bhabha, to frame the migrants’ experiences within these liminal spaces. Through detailed analysis, the article discusses the personal narratives of migrants, their struggles with identity, and the continuous impact of these environments on their psychological well-being. Finally, the article advocates for policy reforms that prioritize human dignity and the integration of migrants’ identities, urging a shift from exclusionary practices to more inclusive and empathetic approaches.

## 2 Review of literature

### 2.1 Non-place

Edward Relph (1976) begins *Place and Placelessness* by emphasizing the primary significance of place to human life: “To be human is to live in a world that is filled with places: to be human is to have and to know your place” (p. 1). Yi-Fu Tuan (1977) makes a similar claim in his seminal book *Space and Place: The Perspective of Experience* when he yokes ‘a sense of place’ to the human need for home: “Space and place are basic components of the lived world; we take them for granted” (p. 3). Other theorists like Cresswell (2015) also valorize human agency in the creation of place by defining it as space that is encountered and transformed through human perceptions, knowledge, and memories (Gebauer et al., 2015). However, De Certeau (1984) offers a critical counterpoint by distinguishing place as static, stable, and ordered from space, which he views as a dynamic canvas for individual freedom and creativity. This tension forms a foundation for understanding migrant spaces, which often lack the rootedness that characterizes traditional places.

In this context, Marc Augé (1995) introduces the concept of the non-place, referring to transitory, anonymous spaces such as airports, detention centers, or highways. These spaces do not support identity, memory, or meaningful relations. They are created through processes of supermodernity and are characterized by functional design, solitary presence, and a lack of communal belonging. They differ from anthropological places, which are rich in history and shared meaning. Augé’s earlier ethnographic work helped develop this distinction by contrasting spaces of cultural continuity with those defined by disconnection and impersonality (Korstanje, 2024; Purdy, 2005).

Bauman (2000) expands on this by describing how liquid modernity leads to social fragmentation and erodes fixed identities. Migrants, especially, face continual disorientation, caught between instability and the loss of community. These conditions generate a lasting psychological sense of being in-between. The absence of rooted place becomes not only a spatial disruption but also an emotional one.

Gebauer et al. (2015) describe this experience as a new structure of feeling. It is marked by discomfort, estrangement, and the erosion of familiarity. Such conditions are frequently depicted in literature and visual narratives about migration. Non-places represent more than physical spaces. They symbolize the condition of being uprooted, unseen, and unacknowledged within systems of modern movement.

Augé (1995) also clarifies that non-place and place can coexist. He writes, “It is possible to think that the same place can be looked upon as a place by some people and as a non-place by others, on a long-term or a short-term basis” (p. 37). This statement highlights that spatial meaning is not fixed. For migrants, the same space may offer temporary safety but never become a source of identity or belonging. This fluidity reflects the unstable, shifting relationship migrants often have with their environments.

### 2.2 Unhomeliness

In the context of postcolonial theory, Homi Bhabha’s concept of unhomeliness provides a critical lens through which to examine the psychological and emotional dislocation experienced by individuals in migration, as well as their navigation through liminal spaces (Pataki, 2018). This concept challenges traditional boundaries between public and private life, providing a critical framework to understand identity crises in the contemporary world. According to Bhabha, ‘home’ is understood as a site of stable identity, a place where one belongs and is recognized (Akbar and Parvaneh, 2016). However, Bhabha (1994) defines unhomeliness as “an estranging sense of the relocation of the home and the world,” where the boundaries between self and other, and between public and private, become blurred. This leads to a simultaneous sense of being at home and not at home, a condition that affects personal and collective identity. As Tyson (2006) explains.

Unhomeliness is an emotional state: unhomed people do not feel at home even in their own homes because they do not feel at home in any culture and, therefore, do not feel at home in themselves. (p. 421).

Unhomeliness also connects with Bhabha’s broader idea of hybridity, which posits that identity is never fixed but always in flux. As individuals move between cultures, they create hybrid identities that resist rigid binaries such as self and other, or home and away (Bhabha, 1994). This process of cultural interaction, transformation, and negotiation produces complex forms of identity that challenge essentialist definitions. As Bhabha explains through the idea of the “third space,” migrants often inhabit an in-between position where they do not fully belong to either culture. This liminal state generates feelings of disorientation and makes it difficult to reconcile a stable self-concept (Bhabha, 1994; Kalua, 2009).

The experience of unhomeliness is also closely associated with the concept of the uncanny, which Bhabha adapts from Freud’s psychoanalytic theory. Freud defines the uncanny as a disturbing feeling that arises when something familiar becomes strange. Bhabha builds on this by arguing that the uncanny can also be internalized, so that individuals experience alienation not only from the external environment but also from themselves (Bhabha, 1994). This internal division complicates identity further, as the individual struggles to distinguish between what is self and what is other (Fotouhi, 2010).

Bhabha’s theory contests the notion of fixed cultural identities. In *The Location of Culture* (1994), he argues that unhomeliness emerges when individuals must occupy multiple, often conflicting, cultural positions. This condition leads to a fragmented sense of self and a lack of belonging in both one’s place of origin and the adopted society. Soi (2018) supports this by emphasizing the importance of “borderlines” where cultures intersect, suggesting that these transitional spaces foster fluid, hybrid identities rather than static ones.

Julia Kristeva’s concept of the uncanny contributes an important dimension to this discussion. She builds upon Freud’s notion by suggesting that the uncanny is not only an external phenomenon but also a psychological experience of alienation from one’s own self (Kristeva, 1991). This perspective corresponds to Bhabha’s interpretation of unhomeliness, where individuals feel disconnected from both their past and present cultural contexts. The uncanny, as Kristeva describes it, involves “the return of the repressed,” a realization that what is most familiar can simultaneously become the most unsettling. For migrants, this often results in a persistent state of psychological disorientation.

## 3 Method

This study employed a qualitative research methodology rooted in thematic analysis (Braun and Clarke, 2019). The approach was designed to examine how the documentary series’ *Living Undocumented* and *Stateless* represent migrant experiences through spatial and psychological themes. The theoretical lens was guided by Marc Augé’s concept of non-place and Homi Bhabha’s theory of unhomeliness, with a focus on the portrayal of trauma, identity, and displacement across visual, auditory, and narrative dimensions. By analyzing character arcs, spatial settings, dialog, and visual motifs, the article situates these series’ within broader academic discussions on identity, displacement, and the emotional geographies of migration.

### 3.1 Materials

The materials selected for analysis were the Netflix documentary series *Living Undocumented* (2019), consisting of six episodes with runtimes between 38 to 45 min each, and the Australian drama series *Stateless* (2020), which contains six episodes of approximately 50–55 min each. *Living Undocumented*, directed by Aaron Saidman and Anna Chai, follows the lives of eight undocumented immigrant families in the United States. *Stateless*, created by Cate Blanchett, Tony Ayres, and Elise McCredie, depicts intersecting narratives in an Australian immigration detention center. Both series were viewed in full on the Netflix streaming platform. All episodes were considered for analysis to preserve the coherence of the narratives and capture recurring visual and thematic patterns.

### 3.2 Procedure

Thematic analysis was used as the primary method of data analysis to identify patterns of meaning across narrative, spatial, and emotional representations (Braun and Clarke, 2019). Both series were watched multiple times. Each viewing pass served a specific purpose: the first established narrative context, while subsequent viewings focused on identifying spaces that qualify as non-places (e.g., detention centers, courtrooms, temporary housing) and moments of unhomeliness (e.g., identity crises, emotional estrangement, cultural displacement). Dialogs were cross-checked using subtitle tracks to ensure transcription accuracy. Cinematographic techniques such as camera angles, lighting, mise-en-scène, and sound design were also analyzed for their contribution to the emotional atmosphere of each non-place or unhomely experience. These elements were examined using a framework organized around core themes of place, identity, trauma, and belonging. Particular attention was given to the portrayal of institutional spaces and their impact on characters’ psychological states, enabling a multi-modal analysis of both visual and verbal representations of trauma.

## 4 Findings and analysis

### 4.1 The psychological and social dynamics of non-places

#### 4.1.1 Depiction of non-places in *Living Undocumented*

*Living Undocumented* (2019) brings to light several non-places that exemplify Marc Augé’s definition: spaces integral to supermodernity but lacking sufficient meaning to be regarded as sites of identity, relation, or history (Augé, 1995). The series follows the trajectories of multiple undocumented individuals as they move through settings shaped by institutional regulation and emotional detachment. These are not merely locations; they are lived environments where the fragility of legal status translates into psychological disorientation. The spatial and emotional registers captured in the documentary series reflect the instability and invisibility that define the undocumented condition.

Augé’s concept of non-places, including spaces like airports, courtrooms, and transit zones, is marked by absence: the absence of memory, of interpersonal connection, and of recognition (Augé, 1995). In the series, these non-places appear not just as backdrops but as dynamic forces that dislocate and define subjectivity. The series does not simply represent these spaces from the outside. It places the viewer inside the emotional field that these spaces produce, through testimonies, silences, and gestures that resist abstraction.

One of the most affectively charged non-places in the series is the detention center. These spaces, designed for management and control, reduce individuals to anonymous figures processed by bureaucracy. To Augé, what comprises the experience of the ‘non-place’ is exactly this uniform practice of following predetermined procedures, often communicating with a counterpart which is a machine or a person wearing a uniform and fulfilling a job function, rather than communicating with persons who are perceived as unique fellow human beings. Thus, by non-places, he means places that facilitate significant aspects of modern life, but do not allow for their user to satisfy important human needs (Gebauer et al., 2015, p. 5).

The documentary reveals the harsh realities faced by detainees, many of whom are incarcerated for extended periods while awaiting deportation or legal resolution. In the fifth episode of the documentary, an immigrant named Miguel describes the holding cells in detention centers, colloquially known as ‘freezer’ or ‘ice box’ (Saidman and Chai, 2019). His words do not serve as illustration but constitute the very analysis. It is through his embodied knowledge that we come to understand what Augé theorizes. These lived conditions—cold, silence, repetition—are not generic markers but personal experiences that define a specific geography of marginalization.

The documentary series emphasizes this transformation through its visual composition. Faces are framed in isolation, surrounded by empty, impersonal walls. The sterility of the architecture is matched by the system’s failure to see the individuals it contains. Within these dehumanizing conditions, the series listens carefully. It allows silence to linger after a testimony, resists editorial summary, and follows the subjects through moments of doubt or breakdown. These are not narrative devices. They are affective arguments, pointing toward the toll of spatial and legal erasure. Merriman’s (2004) insight that non-places lack rootedness becomes legible not because the series explains it, but because the migrants’ experience performs it (p. 145).

The courtroom emerges as another non-place. Here, power is exercised through translation and delay. In one scene, the quiet whisper of an interpreter to a visibly anxious parent underscores how legal processes displace voice. These interactions are not shown as errors or failures; they are the norm. The law appears in the series not as a structure misunderstood by migrants, but as one that deliberately keeps them outside. Through such framing, the documentary series makes visible how legal non-places fragment personhood, converting complex lives into brief procedural appearances.

Temporary housing is also portrayed not as shelter but as suspended habitation. These rooms, often shared or minimally furnished, reflect the impermanence of life on the margins. The series does not merely show these interiors. It listens to what they prevent. Absences become audible through off-screen monologs, as when Pablo expresses uncertainty about ever being allowed to live with stability (Saidman and Chai, 2019, A Family Torn Apart, 29:40). His story is not exceptional. It is positioned as part of a systemic condition, where spatial instability mirrors a wider erasure of rights and recognition.

Awa’s account in episode six, where her father faces deportation to Mauritania, a country that treats him as less than human, disrupts any idea that migration follows a clear path or resolution (Saidman and Chai, 2019, Home Sweet Home). What is called “home” in this context is emptied of protection, dignity, and belonging. It becomes a place of fear, a site where safety cannot even be imagined. The documentary does not soften this reality or frame it for comfort. Her words carry the raw weight of fear, speaking from a place where theory cannot reach. The series does not interpret these voices from outside. It listens. It builds its understanding from within the horror that these stories lay bare.

By centering migrant voices as the starting point for spatial analysis, *Living Undocumented* reconfigures the concept of non-places from an abstract framework into a lived cartography. The series does not simply apply theory. It shows how theoretical insight grows from accumulated, embodied experience. Migrant testimonies are not just present in the narrative; they are the method and the argument. Through them, the documentary series engages deeply with the emotional, legal, and spatial dimensions of exclusion, offering a grounded account of how displacement becomes a condition rather than a moment.

#### 4.1.2 Portrayal of non-place in *Stateless* (2020)

“Excuse me. I do not belong here.”

“No, you do not.”

“How do I get out of here?” (McCredie and Blight, 2020, Incognita, 3:10).

The Australian television series *Stateless* (2020) offers a compelling exploration of migration, detention, and the psychological alienation experienced by individuals within immigration detention centers. The narrative unfolds within a liminal space that exists outside of conventional spatial, legal, and emotional boundaries. *Stateless* centers on the Barton Detention Centre, a desert-bound institution that serves as the central setting. This facility operates as a non-place in the sense that it is physically remote, emotionally vacant, and socially disconnected. The surrounding desert amplifies the psychological desolation of the detainees. Unlike *Living Undocumented*, where non-places are dispersed and varied, *Stateless* maintains a concentrated focus on a single carceral site, which enables a deeper engagement with how spatial enclosure and institutional power reshape identity and mental well-being.

Merriman (2004) argues that non-places often produce non-persons, individuals stripped of the basic social determinants necessary for recognition by the state and society. In *Stateless*, this erasure is enacted through bureaucratic processes, institutional routines, and impersonal environments that reduce people to case numbers and administrative problems. The center is not a neutral backdrop but a mechanism of dehumanization. Unlike spaces such as airports or shopping malls, which allow movement and identity assertion through transactions, detention centers function without any reciprocal engagement. Merriman (2004) notes that in these abject spaces, “the contract is null and void” (p. 148). At Barton, detainees are subject to decisions over which they have no control and with which they cannot negotiate. This dynamic is illustrated in the opening episode when Clare Kowitz, the facility’s new manager, describes the center as a “world-class border protection program,” while the visual framing of fences, surveillance towers, and controlled zones contradicts this claim (McCredie and Blight, 2020, The Circumstances in Which They Come). Javad, a detainee, asks: “You take me to your prison? You tell me, what is my crime?” (McCredie and Blight, 2020, Run Sofie Run, 25:27). His words foreground the tension between official language and lived experience, a recurring theme throughout the series.

One of the most visible markers of non-place in *Stateless* is the systematic reduction of personal identity. Ameer is assigned the label “TOR-076,” stripping him of his name, history, and relationships. Augé (1995) writes that individuals in non-places are meant only to pass through and are denied a sense of belonging. Clare’s directive to isolate “ringleaders” by saying, “empty it if you have to” (McCredie and Blight, 2020, Panis Angelicus, 24:10), illustrates how bureaucratic language furthers this depersonalization. These interactions do not merely represent institutional power but reveal how language itself becomes complicit in producing erasure.

The disruption of time is another significant feature of non-places. Detainees in *Stateless* are caught in indefinite cycles of waiting, with no knowledge of when or whether they will be released. Mina, Ameer’s daughter, questions why they are forced to wait when no answers are given (McCredie and Blight, 2020, The Right Thing). Sofie, misidentified and wrongfully detained, repeatedly asks for clarification, noting that she was meant to be deported to Germany instead. Her confusion reflects how detention centers fracture individuals’ perception of time and place, leaving them suspended between past and future with no clear trajectory.

This sense of temporal and emotional suspension is reinforced through repetition. In one scene, detainees bang on fences while protesting the food, with one exclaiming, “Nobody should be fed this. It is for animals” (McCredie and Blight, 2020, Panis Angelicus, 17:26). The monotonous routines, from the daily meals to the barred communication with the outside world, intensify the experience of unhomeliness. The show does not just depict these routines from a distance; it brings the audience into the sensory and emotional realities of these patterns, showing how survival replaces living.

Ameer’s plea for recognition becomes a crucial moment of resistance. “I am a schoolteacher, a father, a man of faith. Why cannot you see all of that when you look at me?” (McCredie and Blight, 2020, Panis Angelicus, 38:45). His words demand that the viewer, and the system, acknowledge a full person rather than a file. These scenes do not merely support theoretical claims about space and identity; they generate theory through the embodied experience of erasure and refusal. The documentary style of the series, grounded in interviews and reports, adds further resonance to these expressions, suggesting that these dramatized narratives emerge from actual testimonial structures.

The series also attends to how dehumanization affects not only detainees but also those who enforce institutional rules. Clare and Cam, both staff members, grapple with the ethical implications of their roles. Clare’s eventual disillusionment is expressed not through grand statements but through small acts of hesitation, silence, and reflection. Her uncertainty illustrates how non-places do not simply oppress; they implicate and destabilize all who operate within them. The non-place in *Stateless* is not an abstract idea; it is a lived condition, documented through repetition, silence, refusal, and testimony.

#### 4.1.3 The psychological impact of non-places: fear and trauma

Migrants frequently face distinct risk factors for mental health issues, primarily due to their exposure to stressful and traumatizing experiences. These include racial discrimination, urban violence, mistreatment by law enforcement, forced separation from their families, detention or confinement, and the threat or reality of deportation (Bustamante et al., 2017). The psychological toll of living in non-places is evident throughout both documentaries, as many of the migrants featured experience heightened levels of anxiety, depression, and trauma. For some, the constant fear of deportation creates a pervasive sense of instability, exacerbating the already precarious nature of their existence. The constant fear of deportation becomes all-consuming, as migrants are trapped in a cycle of worry and uncertainty. The psychological landscape of *Living Undocumented* is further complicated by past traumas, which are often compounded by the instability of living in non-places. Migrants like Alejandra, who recounts a traumatic robbery from their past, find themselves haunted by both their experiences of violence and the existential threat posed by their undocumented status. The emotional and psychological weight of living in non-places is thus amplified by the compounded traumas of violence, displacement, and the persistent threat of being torn from one’s family.

*Living Undocumented* demonstrates how non-places shape not only the physical experiences of migrants but also their emotional and psychological identities. The dehumanizing nature of detention centers, courtrooms, and temporary housing serves to erode the sense of self, while the constant threat of deportation reinforces the migrant’s status as a “borderline” individual, neither fully part of the community they inhabit nor fully excluded from it. These non-places create a liminal space in which the migrant is trapped, caught between competing national, legal, and personal identities. The documentary, through its intimate portrayal of individual lives, powerfully illustrates the impact of these spaces on the broader migrant experience. Non-places, in this context, do not merely represent physical spaces of transit or bureaucratic process. Instead, they are fundamental to understanding the alienation, fear, and trauma that define the lives of undocumented individuals.

Those non-places that delineate the margins rather than the articulating nodes of society – slums, refugee camps, tent towns, clandestine detention centers – can suspend their dwellers in a long-term or even semipermanent state of transience and negation. For pavement dwellers, slum residents, refugees, and numerous others, dispossession becomes a significant part of their identity and relations (Merriman, 2004, p. 148).

In *Stateless*, the psychological toll of inhabiting a non-place is evident in both detainees and staff. For detainees, the constant surveillance, lack of autonomy, and indefinite detention result in deteriorating mental health. Sofie’s breakdown is emblematic of this, as her attempts to retain her sense of self collapse under the weight of systemic oppression and unresolved trauma. “I just want to stay out here, in the fresh air,” she pleads (McCredie and Blight, 2020, Panis Angelicus, 31:19), her desperation highlighting the suffocating nature of Barton. The intersection of gender with experiences of displacement and confinement is especially visible in the portrayal of Sofie, whose mental health crisis becomes both a cause and a consequence of her detention. While her story is based on the real-life case of Cornelia Rau, the series does more than narrate a biographical account. It foregrounds how institutional systems frequently pathologize women who resist normative roles or struggle with trauma. Sofie’s identity is constantly questioned, from her initial rejection by the dance company to her dehumanization within the detention center. Her experience reveals how gendered assumptions about vulnerability, irrationality, and deviance interact with carceral logics to justify indefinite detention (Canning, 2017). Unlike male detainees, whose struggle often centers on bureaucratic erasure or family separation, Sofie’s journey illuminates the gendered politics of mental illness and credibility.

Moreover, the experiences of Mina, Ameer’s daughter, offer a glimpse into how children are not shielded from the psychological weight of statelessness. Her confusion and frustration with waiting, her desire for clarity and routine, and her attempts to comfort her father all point to how childhood is reshaped in the context of confinement. These dynamics are not presented as peripheral. They are central to the series’ critique of systems that disregard the specific needs and rights of women and children, whose experiences of statelessness are mediated not only by space and legality but also by gendered expectations of care, behavior, and resistance.

Staff members, too, are affected by the moral ambiguities of their roles. Cam Sandford, a guard, oscillates between enforcing rules and questioning their ethical implications. His guilt over betraying Janice and aiding in the recapture of escapees manifests in aggressive outbursts and a strained relationship with his family. “What was I supposed to do?” he asks, torn between loyalty to his sister and his duties (McCredie and Blight, 2020, Panis Angelicus, 13:22). Cam’s breakdown demonstrates how even those in positions of power are not immune to the corrosive effects of non-places.

### 4.2 Depiction of unhomeliness in *Living Undocumented* and *Stateless*

#### 4.2.1 Personal narratives of unhomeliness in *Living Undocumented*

In *Living Undocumented*, the intense feelings of unhomeliness articulated by Homi Bhabha become palpable through the lived experiences of the featured individuals, as they navigate the liminal spaces of their existence within the United States. Bhabha describes unhomeliness not as homelessness but as a profound disruption of the domestic familiar, where the private and public spheres intermingle and produce a feeling of alienation and displacement (Bhabha, 1994). This documentary series exemplifies how the immigration system, with its arbitrary divisions and enforcement of a ‘zero tolerance’ policy, catalyzes this sense of unhomeliness, especially in environments that should be places of refuge but instead become zones of exclusion.

The opening story of Luis, Kenia, and their son Noah, set in a nondescript Kansas City parking lot, embodies this dislocation. The parking lot functions as both a literal and symbolic threshold. It is a space of waiting that reflects their uncertain legal status. As they anticipate Kenia’s release from detention, the ordinary setting transforms into a site of anxiety and emotional exposure. Their private suffering becomes a public scene, entangled with the impersonal reach of state power. When Kenia is not brought out and Luis risks arrest just to say goodbye, the moment captures the essence of Bhabha’s unhomely. This supposedly neutral public space becomes alien, unsettling, and marked by fear (Aman and Dahlstedt, 2021).

This experience intensifies with Alejandra, a military spouse and mother of three, who faces deportation after over two decades in the United States. Her home, once a site of belonging, becomes saturated with fear and instability. As she prepares to leave, the domestic space shifts into a scene of emotional rupture. It is no longer private or safe. Packing 21 years of life into a suitcase while cameras follow her every move, Alejandra’s experience reflects a home turned inside out. It becomes a site of personal loss, staged before a public audience. This reversal mirrors Bhabha (1994) notion of the home as no longer a sanctuary, but a contested space between belonging and exclusion.

The documentary uses cinematic techniques to express the emotional dislocation of unhomeliness. Close-ups of personal items and domestic spaces highlight the emotional stakes, while their contrast with the sterile visuals of ICE offices and courtrooms underscores institutional coldness. Diegetic sounds, like echoing footsteps and hushed legal conversations, heighten the sense of isolation and in-betweenness. Rather than aestheticizing suffering, the series reflects what Mavelli (2017) calls an “ethics of appearance,” where visibility fosters recognition and responsibility. Its tight framing, muted colors, and restrained tone resist sentimentality, portraying migrant pain without reducing it to spectacle. Through these choices, the documentary reveals unhomeliness not just as emotional trauma but as a condition shaped by systemic neglect.

*Living Undocumented* also explores the public dimension of unhomeliness. The documentary reveals how these private struggles are politicized, showcasing the broader societal and governmental forces at play. Discussions by legal experts and activists provide a macro perspective on the issues, linking personal stories with national policies. This framing not only highlights the systemic nature of the problems faced by the individuals but also situates their experiences within the larger discourse of immigration, identity, and belonging.

The interplay between the personal narratives and the public discourse creates a powerful commentary on the state of being unhomed. As Bhabha suggests, unhomeliness is a complex construct where the personal and the political intersect, where the home becomes a site of unbelonging, and where individuals live the paradox of being visible yet invisible, included yet excluded (Bhabha, 1994).

#### 4.2.2 Unhomeliness and psychological displacement in *Stateless*

Alongside the erasure of identity, *Stateless* vividly portrays the concept of unhomeliness, particularly through the character of Sofie, an Australian citizen who finds herself wrongfully detained in the immigration center. Although Sofie legally belongs to Australia, she becomes a victim of the very system that is supposed to protect her, exposing her to the psychological dislocation and alienation that defines unhomeliness. Homi Bhabha’s theory of unhomeliness describes the condition of feeling alienated even within one’s own country, as individuals experience profound estrangement from their own identity and sense of belonging (Bhabha, 1994). Sofie’s journey illustrates how legal belonging is not always synonymous with a psychological or emotional connection to one’s place of residence.

Sofie’s internal struggle is highlighted when she interacts with Pat, a cult leader within the detention center. Pat tells Sofie, “How could I let you in, with your dirty, negative energy?” (McCredie and Blight, 2020, The Right Thing, 42:28). This rejection of Sofie from an individual who is himself a victim of the system underscores her experience of unhomeliness. Despite her legal status as an Australian, Sofie is rendered an outsider within the very system that should have provided her with security (Tindale, 2023). Her psychological unraveling, marked by her inability to reconcile her fractured identity within the context of the detention system, reflects Bhabha’s concept of unhomeliness: an alienation not from a physical place but from one’s sense of belonging and identity.

Moreover, Sofie’s unhomeliness is compounded by the cultural and social dynamics within the detention center, where she is treated with suspicion and distrust. In a conversation with Clare, a social worker. Sofie reflects on how her identity is being degraded as she comments on being treated as if she were one of the others. This statement reveals Sofie’s acute awareness of the social and psychological implications of being confined within a non-place that does not recognize her identity. While Sofie’s body remains in Australia, her mind and sense of self are displaced into a state of alienation and psychological exile.

*Stateless* also employs visual storytelling to reinforce the themes of non-place and unhomeliness. The sterile, institutional design of the detention center is highlighted through wide shots that emphasize the emotional and physical isolation of the detainees. The lack of personal space and the monotonous, uniform architecture of the center contribute to the sense of claustrophobia and psychological confinement experienced by the characters. The visual style emphasizes monotony and restriction through recurring imagery of fences, surveillance cameras, and the oppressive symmetry of detention center layouts. As the camera lingers on the impersonal, barren spaces within the detention center, it visually conveys the emotional desolation of the characters who are trapped in this non-place.

In contrast, flashbacks to the characters’ lives before their detention are often depicted with warmer tones and more dynamic compositions, emphasizing the emotional and personal connections that have been lost. These contrasts highlight the psychological rupture between remembered belonging and current displacement. The auditory landscape is equally significant. The incessant hum of fluorescent lights, the echo of metal doors slamming shut, and the omnipresent announcements on loudspeakers create a soundscape of control and alienation. These auditory cues contribute to the oppressive atmosphere, keeping both characters and viewers in a constant state of unease.

## 5 Discussion

*Living Undocumented* and *Stateless* extend beyond narrative representation to evoke the lived realities of displacement, alienation, and resistance. Through their distinct formats—documentary and dramatized fiction—these series foreground the spatial and psychological dimensions of migrant life, offering textured illustrations of *non-place* and *unhomeliness* as theorized by Marc Augé (1995) and Homi Bhabha (1992). *Non-places*, such as detention centers, courtrooms, and temporary shelters, emerge not only as physical sites but also as architectures of anonymity, surveillance, and control. They regulate movement, suppress identity, and enforce a condition of liminality. *Unhomeliness*, in contrast, captures the inner rupture experienced when the space of “home” becomes estranged. It refers to the migrant’s sense of being caught between cultures, dislocated within their own body and memory.

These two frameworks, though distinct, coalesce in the series to illustrate how migration is not only spatial but existential. Rather than unfolding in a linear sequence, *non-place* and *unhomeliness* often co-construct each other. Non-places generate the affective condition of unhomeliness by stripping individuals of social belonging and temporal continuity. Detention centers, for instance, do not merely disrupt mobility; they alter the migrant’s relationship to time, community, and self-perception. Conversely, the condition of unhomeliness may persist even beyond formal non-places. Migrants may continue to experience alienation in homes, schools, and neighborhoods when their histories and identities are rendered invisible or incompatible with dominant cultural narratives. In this sense, *non-place* can be understood not as a precondition but as one axis through which *unhomeliness* manifests. The two frameworks overlap across legal, physical, and affective domains, creating what may be termed a compounded dislocation: legal exclusion reinforces spatial marginality, which in turn deepens psychological estrangement.

*Stateless* clearly portrays this dynamic. Legal tribunals and administrative systems do not simply detain; they actively produce illegality and fragmentation. As De Genova (2013) argues, such systems fabricate exclusion by treating personal histories as irrelevant and identities as unstable. Characters like Sofie and Ameer are not illegal by nature; they are rendered so through encounters with opaque and indifferent bureaucracies. *Living Undocumented* similarly avoids reducing migrants to passive victims. The series foregrounds agency and contradiction, resisting the “politics of humanitarianism” critiqued by Ticktin (2016). Migrant voices emerge not as illustrations of theory but as sources of theoretical insight, allowing the affective geography of displacement to speak for itself.

The visual aesthetics of *Living Undocumented* further reinforce this ethical orientation. Rather than aestheticizing suffering, the series adopts what Mavelli (2017) calls an “ethics of appearance,” where representation fosters recognition without objectification. Through unobtrusive editing, ambient sound, and close-up framing, the series maintains dignity and emotional depth. *Stateless* follows a similar approach. Although dramatized, its restraint avoids spectacle and focuses on the psychological toll of indefinite detention, bureaucratic surveillance, and public indifference.

Both series show that migrants inhabit overlapping geographies of loss, hope, and survival. The detention center, the waiting room, and the interview booth become emblematic *non-places* not only in spatial terms but also in how they shape the migrant’s sense of self and time. Yet even within these spaces, the series’ depict moments of care, assertion, and community-building that resist total erasure. These portrayals suggest that *unhomeliness* is not merely endured but can also be confronted, narrated, and, at times, transformed.

*Living Undocumented* is based on direct testimonies of migrants navigating the U. S. immigration system. *Stateless* draws from the documented case of Cornelia Rau and testimonies from former detainees. These are not imagined stories; they are grounded in lived experience and legal archives. As such, these portrayals serve as more than narratives. They perform acts of witnessing. They compel ethical reflection on the legal, psychological, and spatial infrastructures that shape contemporary migrant life.

[Non-place] never exists in its pure form; places reconstitute themselves in it; relations are restored and resumed in it. Place and non-place are rather like opposed polarities: the first is never completely erased, the second is never totally completed; they are like palimpsests on which the scrambled game of identity and relations is ceaselessly rewritten. (Augé 1995, pp. 78–9).

Reflecting on Augé’s assertion that places and non-places are continuously rewritten through interactions, it becomes clear that our societal structures and spaces are not fixed but fluid. They are shaped by the stories we tell, the policies we implement, and the empathy we extend. As such, transforming non-places into spaces of belonging and acceptance is not only possible but essential. This transformation requires a collective reimagining of our social fabrics, woven with threads of inclusivity, understanding, and respect.

*Living Undocumented* and *Stateless* challenge us to rethink the meaning of belonging. They provoke a critical examination of how migration is managed and the role non-places play in our societies. These series encourage us to look beyond the surface to the structural and emotional landscapes that shape human lives globally, advocating for a scholarly and practical reevaluation of how we perceive, and indeed how we construct, the concept of place in our disjointed world. Let these narratives inspire not only reflection but also action in the areas of migration policy and human rights, promoting a world where every individual has a place to call home.

### 5.1 Implications

The analysis of *Living Undocumented* and *Stateless* reveals how non-places and unhomeliness shape not only the conditions of migrant life but also the narratives through which these conditions are understood. For migration scholarship, these findings offer a reminder that affective and spatial dimensions must be placed alongside legal and structural analyses. Bureaucratic spaces like detention centers, immigration courts, and surveillance zones are not only logistical sites but affective architectures that structure how migrants are seen, heard, and held in place. However, these insights must also move beyond academic framing into public policy. If we take seriously the idea that spatial design and administrative routines produce trauma, then a trauma-informed migration system would look markedly different. It would resist the normalization of confinement and instead support policies rooted in stability, dignity, and care. For instance, replacing prolonged detention with community-based alternatives, offering consistent legal and psychological support, and training border and immigration officials in trauma-awareness and cultural competency are not abstract ideals but practical interventions already piloted in parts of Canada and Germany (Steel et al., 2011; Kronick et al., 2011). These reforms reimagine space as reparative rather than punitive, where migrants can begin to reconstruct continuity, autonomy, and identity. Furthermore, visual media like the series examined here play a critical role in shaping the collective imagination of migration. When series like *Living Undocumented* center migrant voices in their complexity, including moments of contradiction, resilience, and agency, they reflect what Mavelli (2017) describes as an ethics of appearance. In this approach, visibility enables recognition and accountability without reducing individuals to passive representations. These forms of storytelling do not replace policy reform. These media interventions are not substitutes for policy change, but they can enable a cultural shift in how migration is narrated, politicized, and ultimately governed.

### 5.2 Limitations and future directions

While this paper foregrounds the frameworks of non-place and unhomeliness, it does so with the recognition that these are not exhaustive or universally explanatory categories. Their strength lies in theorizing estrangement and structural precarity, but they may overlook the everyday strategies of adaptation, resistance, and relational repair that many migrants practice. This analysis does not assume their totalizing validity but rather uses them to foreground specific dimensions of migrant experience. Alternative frameworks such as resilience (Gatt et al., 2020; Papadopoulos, 2007), social capital (Lőrincz and Németh, 2022; Portes, 1998), and cultural adaptation (Benjamin et al., 2025) offer valuable correctives. These perspectives highlight how migrants forge networks, reassert identity, and rebuild belonging within and beyond non-places. Future research would benefit from placing these frameworks in dialog, balancing critical analysis of spatial alienation with attention to migrant agency and recovery. Additionally, this study engages with two Anglophone, high-production media texts from the United States and Australia. While these were chosen for the diverse migrant experiences they depict, their framing remains rooted in Western perspectives. Future research could expand on this by including media created within or by Global South communities, allowing for more direct engagement with non-Western perspectives and forms of self-representation. Lastly, incorporating field-based research such as interviews, oral histories, or community-based participatory methods would deepen the empirical grounding of future analyses. This would allow theoretical insights to emerge more directly from lived experience and support a more accountable and ethically responsive approach to migration scholarship.

## Data Availability

The original contributions presented in the study are included in the article/supplementary material, further inquiries can be directed to the corresponding author/s.
